# A diagnostic algorithm for evaluating cases of potential macrocyclic lactone–resistant heartworm

**DOI:** 10.1186/s13071-017-2441-9

**Published:** 2017-11-09

**Authors:** Andrew R. Moorhead, Christopher C. Evans, Ray M. Kaplan

**Affiliations:** Department of Infectious Diseases, College of Veterinary Medicine, University of Georgia, 501 D. W. Brooks Drive, Athens, GA 30605 USA

**Keywords:** Heartworm, *Dirofilaria immitis*, Macrocyclic lactone, Resistance

## Abstract

**Background:**

The emergence of macrocyclic lactone resistance in canine heartworm poses a substantial threat to what is currently the only effective, FDA-approved available method of prevention. Further study of the biotypes is necessary to understand the mechanism of resistance and evaluate novel prevention options. Identifying cases of drug-resistant infection remains problematic, however, especially when poor compliance and insufficient testing are concerns. Furthermore, a definitive demonstration of resistance requires experimental infection and treatment, which is prohibitively costly.

**Methods:**

With the aim of identifying likely cases of macrocyclic lactone-resistant heartworm and preventing their continued spread, we describe an algorithm for determining the likelihood of drug resistance and appropriate treatment strategies for each case.

**Results:**

This algorithm relies on the microfilarial suppression test (MFST), which has been used previously as an efficient and discrete measure of suspected resistance. By standardizing this method in a format that is readily available to practitioners, it could become possible to preliminarily survey the emergence and spread of resistance.

**Conclusion:**

Heartworm isolates identified through this method can be used in research to better understand macrocyclic lactone resistance so prevention strategies can be adapted.

## Background

The only FDA-approved available method of preventing the development of adult heartworms (*Dirofilaria immitis*) in dogs and cats is the compliant administration of products containing macrocyclic lactone (ML) drugs [1]. These products are extremely effective against third-stage larvae (L3) and up to 30-day-old fourth-stage larvae, with a label efficacy of 100%. Recently, ML resistance in heartworm has been suspected in clinical cases, as well as documented in experimental settings [1–4]. Most, if not all, credible cases of possible ML resistance in heartworm have been diagnosed in states bordering the lower Mississippi River, referred to as the Mississippi Delta region.

In Atkins et al. [5], the authors examined 319 lack of efficacy (LOE) cases from the Mississippi Delta region of the United States using the Window of Infection tool (http://www.heartwormedu.com/). The authors concluded that in 98.7% of the cases there was insufficient product purchased, or product was being shared between multiple dogs in the household, suggesting that these cases likely were due to noncompliance in administration. However, it should be noted that in some of these cases the lack of compliance was outside of the window of infection. Nevertheless, in 1.7% of the cases (5/319), the cause of the LOE could not be attributed to noncompliance or extenuating circumstances. One possible explanation is that these represent cases where heartworms were resistant to the ML [5].

Currently, no point-of-care clinically applicable tests for resistance in heartworm are available. Furthermore, there are no validated laboratory tests for detecting resistance in heartworm. Thus, definitive diagnosis in suspected heartworm resistance cases remains impossible in a clinical setting, and the only means presently available to prove resistance is to perform a controlled efficacy study in a laboratory environment [2, 3]. Briefly, one must collect microfilaremic blood from the suspect case and then feed that blood to mosquitoes. After 14 days the infective L3 are harvested from the mosquitoes and injected into a dog. Thirty days later a dose of ML is administered followed by four, five, or eight additional monthly doses [2, 3]. If the dog subsequently becomes antigen-positive, then the conclusion is that the worms in the suspect case were indeed an ML-resistant biotype. Clearly, this is an extremely complicated and expensive procedure, making it useful only for research purposes. Consequently, not only do we currently lack the ability to diagnose cases of resistance definitively at the clinical level, we also are unable to determine the prevalence and distribution of resistance in heartworm. Recently, some progress has been made in identifying genetic markers that may be associated with resistance [2, 6], however it remains unknown if this work will lead to a useful diagnostic test.

### So what is a veterinarian to do when presented with a case of suspected resistance?

Lacking a diagnostic test for resistance in heartworm, is there anything a veterinarian can do when presented with a case of suspected resistance? One approach is to use a surrogate measurement for drug efficacy. There is a substantial body of literature demonstrating rapid (within 4–6 h) and profound reductions in microfilaria (MF) levels following microfilaricidal doses of ML in dogs infected with known ML-susceptible heartworms [7]. Additionally, dogs infected with heartworms usually become amicrofilaremic following several monthly doses of ML. Thus, one would expect a dog on compliant ML prophylaxis to be free of MF unless the dog is infected with a drug-resistant biotype. In contrast, to our knowledge, in every case of proven ML resistance the dog was microfilaremic at the time of diagnosis despite receiving monthly treatments with ML drugs. These observations provide an opportunity to develop a clinical test based on the measurement of MF levels both before and after a microfilaricidal dose of ML. This test, referred to as the MFST (microfilarial suppression test), was first proposed by Geary et al. [7]. Based on historical data indicating the expectation of a rapid and profound decrease in levels of MF, Geary (2011) proposed that reductions in MF of <75% following a microfilaricidal dose of ML are suggestive of resistance. If reduction in MF numbers is >75%, the case is unlikely to represent ML resistance.

## Methods

**Fig. 1:**
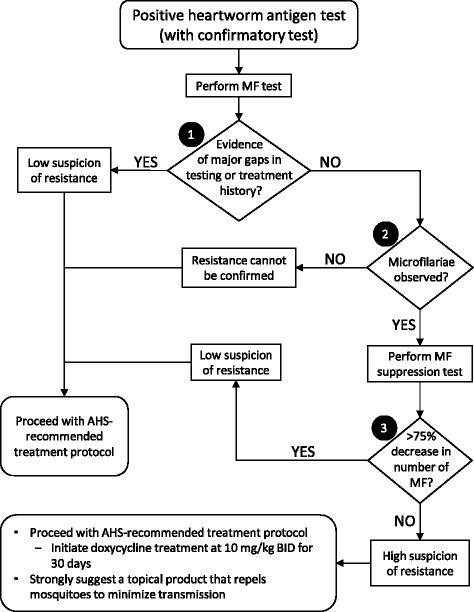
Diagnostic algorithm for evaluating cases of potential macrocyclic lactone-resistant heartworm

Although it is possible that new data might be produced that contradict this assertion, current evidence suggests that the MFST is the most practical and accurate method for determining whether a heartworm case is ML-resistant. Consequently, veterinary practitioners could benefit from a diagnostic algorithm for evaluating suspected ML resistance cases; and, in this article, we detail a decision tree for such evaluation (Fig. 1).

## Results

### A decision tree for potential cases of ML resistance

#### Node 1 – Compliance, purchase history, and testing

The evaluation of any case for potential ML resistance begins with a positive result on a commercially available heartworm antigen test (Fig. 1). The first question that must be asked is whether the client is compliant with ML administration and testing. We cannot verify owner compliance of ML dosing unless the veterinarian directly observes the owner administering the drug, or, alternatively, the veterinarian or his/her staff administer each ML dose. Because verification of owner compliance is difficult, the veterinarian must inquire as to the consistency of the client’s ML dosing. Concurrent with this inquiry, the client’s purchase history must be checked, since gaps in the purchasing of preventive would indicate a gap in administration of ML. If ML preventives have not been administered, there are gaps in administration per the owner’s history, or there are gaps in the purchase history, then it is reasonable to assume that the case is not likely due to an infection with a resistant biotype.

The gaps referred to previously are at least 2 months in administration of preventive. For smaller gaps, those less than 2 months, the scenario is different. Specifically, if the owner missed only one or two doses and was consistently compliant at all other times, then the suspicion of resistance would be reasonable, especially when the safety net (ie, reachback) effect is taken into account. The safety net effect is defined as the time period that an animal did not receive heartworm preventive but still did not develop adult heartworms after subsequent administration of ML preventives [8]. In one study when ivermectin was administered at the preventive dose for a period of 1 year starting at 3 or 4 months postinfection with heartworm larvae, it was 97.7 and 95.1% effective, respectively. When milbemycin oxime was administered during a similar time period, the efficacy against 3-month-old worms was 96.8%, whereas efficacy against 4-month-old worms was 41.4% [9]. Similarly, selamectin administered monthly for 1 year was 98.5% effective against 3-month-old worms [8]. Additionally, there are data indicating that a single dose of the topical formulation of 2.5% moxidectin and 10% imidacloprid combined with doxycycline (10 mg/kg BID for 30 days) will kill 3.5- and 5-month-old worms [10].

These data indicate that, while efficacy against immature heartworms may vary for the different ML, there is essentially a grace period in which an otherwise compliant owner could forget to administer a dose of heartworm preventive without significant risk of that gap leading to an infection. Consequently, LOE cases where the owner missed administering only one or two doses are suspicious for resistance and warrant further investigation.

Along with compliance, the other factor that must initially be addressed in the evaluation of resistance is whether there are gaps in heartworm antigen testing. The animal should be tested prior to the start of administration of preventive if over 6 months of age to ensure no infection exists. In the absence of this test, we cannot determine whether the current positive result was due to a recent or previous infection. Furthermore, any test performed within the first 6 months of compliant prophylaxis offers no insight into the resistance status of the infection. In either of these situations, the clinician should have a low index of suspicion that this is a case of resistance.

For cases where there are marked gaps in either history or testing, then the suspicion of resistance is low; and one should proceed with treatment following the American Heartworm Society (AHS)-recommended protocol, which uses three doses of melarsomine dihydrochloride, doxycycline and an ML to clear MF and prevent further transmission (www.heartwormsociety.org).

#### Node 2 – Observation of microfilariae

The current AHS recommendation is to perform an examination for MF in all heartworm-positive cases. If no MF are observed, resistance can neither be disproven nor confirmed. Even if the biotype was ML-resistant, further transmission of resistant worms is not possible, as there are no MF for the mosquitoes to ingest. Without MF, there is no development of L3 and, hence, no transmission. Because resistance can neither be confirmed nor refuted, one should proceed with the AHS-recommended treatment protocol.

If MF are observed, then the MF are persisting in the face of continuous use of ML (even with the minor gaps as explained previously. It is the experience of the authors, and is noted in other published works, that all previous cases of confirmed resistance maintained a microfilaremia even after the administration of ML at microfilaricidal doses [1, 2]. Because MF are present, these cases have a high index of suspicion and the MFST should be performed [7].

#### Node 3 – The microfilarial suppression test (MFST)

As already stated, presently the MFST is the only patient-side test that can adequately predict whether an LOE case has a high or low suspicion of resistance. The three steps of the MFST are as follows:

1. *Perform Knott’s test for quantitation of MF.* The procedure for this test is located at www.heartwormsociety.org. The entire sample should be counted in order to gain the number of MF per milliliter.
2. *Administer ivermectin at 50 μg/kg, or milbemycin oxime at 1 mg/kg, after obtaining blood for the Knott’s test.* It is important to remember that both medications are microfilaricidal at these doses, which causes rapid disappearance (ie, within 4–8 h) of susceptible MF but not of resistant MF. One will not know whether the MF are susceptible; and, therefore, these animals should be treated prior to the administration of ML with diphenhydramine and steroids in order to prevent anaphylaxis due to the rapid death of MF (www.heartwormsociety.org). However, it is important to keep in mind that you would only perform the MFST in a case where the owner is providing apparent compliant prophylaxis. If the infection was acquired prior to the initiation of prophylaxis, or is due to a break in prophylaxis rather than due to ML-resistant heartworms, then there should be no or very few circulating MF, making the MFST unnecessary and anaphylaxis highly unlikely. If the biotype is ML-resistant and the animal has been on compliantly administered preventive, then presumably the rapid death of MF will not occur; thus, there is little risk in performing the MFST. Nevertheless, because of the potential risk of anaphylaxis, the microfilaricidal doses of ivermectin or milbemycin oxime should be administered at the veterinary hospital and the dog observed for 6–8 h post microfilaricide administration.

Topical 2.5% moxidectin and 10% imidacloprid is an FDA-approved microfilaricide, and the risk of anaphylaxis when 2.5% moxidectin and 10% imidacloprid is administered to a microfilaremic dog is less than ivermectin at 50 μg/kg or milbemycin oxime at 1 mg/kg [11, 12]. However, the use of this product in the MFST has never been documented in the literature; and, thus, we lack the data necessary to interpret the results properly following its use. Consequently, until studies are performed and validate its use, we do not advocate the use of topical 2.5% moxidectin and 10% imidacloprid in the MFST.

3. *Perform a second Knott’s test for MF quantification 7 days after the initial test.* The percentage reduction in numbers of MF between the first and second Knott’s tests determines whether the case has a high or low suspicion of resistance. If there is a > 75% decrease in MF numbers between the two tests, then our suspicion of ML resistance will be low. If there is <75%, then our index of suspicion will be high. It should be noted, that historically there will be a 90–95% reduction of MF after use of 50 μg/kg of ivermectin [13]. One could reasonably expect a high degree of variability in this test, and additional contemporary experimental data would be desirable. However, the experiments required to refine this cutoff would be expensive, requiring a large number of dogs of different breeds. Since these experiments will not likely be performed, using published historical data (ie, 75% decrease) is the most practical approach.

For both high- and low-suspicion cases, one immediately proceeds with the AHS-recommended adulticide treatment. It must be emphasized that, at the initiation of this treatment regimen, doxycycline is administered at a dose of 10 mg/kg BID for 30 days. This is important due to the fact that McCall et al. (2014) reported that MF taken from a dog treated with doxycycline developed to L3 in mosquitoes, but that those L3 did not subsequently develop to adult heartworms when used to infect dogs. In other words, the doxycycline essentially limited the infectivity of the L3 derived from the heartworm-infected dog. In conjunction with doxycycline, it is also important to consider a topical product that is labeled to repel mosquitoes. While repellency of mosquitoes is not a substitute for ML heartworm preventives, a mosquito repellent does provide an added layer of protection that is desirable, especially when dealing with transmission of a potentially resistant biotype.

## Discussion

Realistically, all cases of heartworm should be treated using melarsomine, preferably following the AHS guidelines. For this reason, the question could be asked as to whether there is a practical utility to applying this algorithm in a clinical situation. The authors feel that the use of this diagnostic procedure is important, as it allows us, to the best or our current scientific capabilities, to determine which cases are most likely due to resistance and where they are originating. With these data, we can begin to evaluate the potential prevalence and geographic distribution of ML resistance; and veterinarians will be able to judge whether ML-resistant heartworms are circulating in their practice area.

## Conclusion

In the absence of a test that provides conclusive proof of resistance, the algorithm presented here represents a viable tool for clinicians to investigate and report resitance in heartworm cases.

## Abbreviations

LOE: Lack of efficacy
MF: Microfilaria
MFST: Microfilarial suppression test
ML: Macrocyclic lactone
